# Ultrasound-Guided Peripheral Intravenous Line Placement: A Narrative Review of Evidence-based Best Practices

**DOI:** 10.5811/westjem.2017.7.34610

**Published:** 2017-09-11

**Authors:** Michael Gottlieb, Tina Sundaram, Dallas Holladay, Damali Nakitende

**Affiliations:** Rush University Medical Center, Department of Emergency Medicine, Chicago, Illinois

## Abstract

Peripheral intravenous line placement is a common procedure in emergency medicine. Ultrasound guidance has been demonstrated to improve success rates, as well as decrease complications and pain. This paper provides a narrative review of the literature focusing on best practices and techniques to improve performance with this procedure. We provide an evidence-based discussion of preparation for the procedure, vein and catheter selection, multiple techniques for placement, and line confirmation.

## BACKGROUND

After the first reported use of ultrasound for real-time central venous catheter (CVC) insertion was reported in 1984,^1^ ultrasound-guidance progressively became the standard approach for placement, particularly when cannulating the internal jugular vein.^2^,^3^ When used for CVC insertion, ultrasound guidance has led to increased placement success, decreased complication rates, and decreased insertion times.^4^ As ultrasound technology and training have improved, researchers have studied whether the benefits of ultrasound in central venous access would translate to peripheral intravenous line (PIV) placement.

Peripheral intravenous access is the most commonly performed procedure in the emergency department (ED), with 150–200 million PIVs placed annually in North America.^5^,^6^ Unfortunately, diseases frequently encountered in the ED, such as diabetes, intravenous drug abuse, and sickle cell disease, are often associated with difficulty of PIV placement.^5^,^7^ Studies have demonstrated that as many as 8–23% of ED patients meet criteria for difficult venous access.^5^,^8^ Historically, these patients have frequently required “rescue” techniques, such as the placement of an external jugular line or CVC insertion when PIVs could not be obtained by landmark guidance. However, CVCs are associated with much more serious complications when compared to PIVs.^9^,^10^ Complications associated with CVC placement include infections, hemothorax, pneumothorax, arterial puncture, and hematoma formation.^9^ Several studies have found that the incorporation of ultrasound-guided PIV can reduce the need for CVC placement in up to 80% of patients.^11^–^14^

Multiple studies of ultrasound-guided versus landmark-based PIV insertion have demonstrated that the use of ultrasound improves placement success, with the most pronounced effects occurring in those with difficult access.^15^–^25^ While many PIV placements may not necessitate ultrasound, a significant number of patients may benefit from this approach by either reducing the number of PIV attempts or preventing the need for CVC insertion. Therefore, it is important that all providers be comfortable with this application.

This paper provides a narrative review of the literature on all components of ultrasound-guided PIV placement from preparation to confirmation with a focus on best practices and techniques for improved performance with this procedure.

## CRITICAL APPRAISAL OF THE LITERATURE

We performed a search of PubMed for articles published from inception to June 16, 2017. Keywords included “ultrasound,” “peripheral line,” “peripheral iv,” “venous access,” and “vascular access.” Bibliographies of all relevant articles were reviewed for additional studies. The search yielded 2,620 articles, of which 65 articles were deemed to be relevant for inclusion in this review. When supporting data was not available, recommendations were made based upon the authors’ combined experience and opinions.

## PREPARATION

Prior to beginning the procedure, all appropriate supplies should be gathered and any relevant contraindications should be evaluated (e.g., hemodialysis fistula, history of ipsilateral mastectomy or lymph node dissection, etc.). Similar to blind PIV placement, it is beneficial to ask the patient which arm has had a higher rate of successful cannulation in the past. It is important to recognize that there are innate risks related to peripheral vascular access with or without the use of ultrasound. These include infection, bleeding, and damage to adjacent structures (e.g., arteries and nerves). In a study performed by Adhikari et al., there was no increase in infection rates in ultrasound-guided peripheral lines when compared to traditionally placed peripheral lines.^26^

Frazee et al. demonstrated that methicillin-resistant *Staphylococcus aureus* (MRSA) and other clinically-significant organisms were effectively eliminated from the transducer with the use of a quaternary ammonia-based germicidal wipe.^27^ Although chemical disinfectants have been shown to decrease the spread of pathogens, several barrier methods including probe covers and adhesive films (e.g., 3M Tegaderm^TM^) are frequently used to further decrease infection risk. Current data is limited on the efficacy of adhesive films for decreasing the risk of infection, and further studies are needed.^28^ Caution should be used with the application of an adhesive barrier, as some manufacturers recommend against its use based upon concern that it may damage the ultrasound probe’s protective membrane.

To perform the procedure, several supplies are needed. These include a tourniquet, alcohol pads, gauze, normal saline flushes, PIV tubing, PIV catheters, adhesive to secure the line after placement, and sterile ultrasound transmission gel (or alternate sterile gel that can transmit ultrasound waves). The ultrasound machine should be placed on the contralateral side of the bed so that it is in the direct line of sight for the provider. Given the potential for infection transmission, it is important to use sterile ultrasound gel or lubricant during placement.^29^ If available, guidewire-based catheters can be used to increase success of catheter advancement after the vessel is cannulated.^30^ When applying the tourniquet, it should be applied as close to the axilla as possible to increase the degree of venodilation present. Sometimes the addition of a second tourniquet or a blood pressure cuff inflated to 150 mm Hg may be needed to ensure sufficient venous distension.^31^

## BEST PRACTICE RECOMMENDATIONS

1. Standard PIV placement and cleaning procedures should be followed. Sterile ultrasound gel should be used during placement.
2. There is limited evidence with respect to the benefit of probe covers and adhesive barriers. Manufacturer recommendations should be followed when using adhesive barriers.

## VEIN CHARACTERISTICS

**Figure 1:**
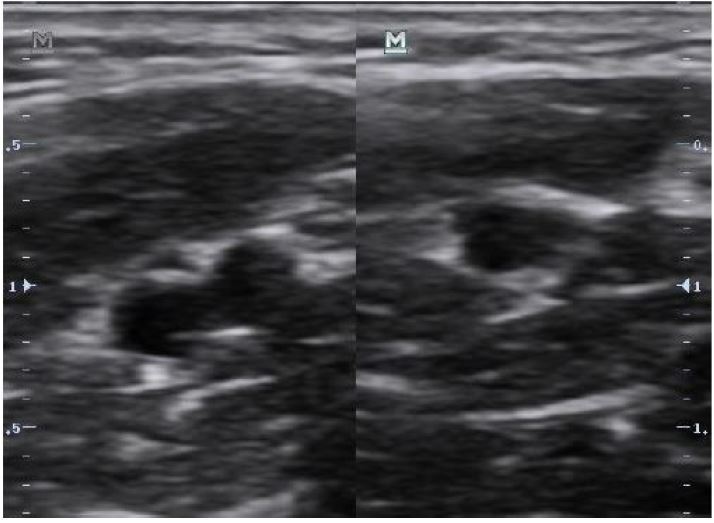
Differentiation of vein from artery using compression. The left image demonstrates both artery and vein. The right image demonstrates only an artery.

**Figure 2:**
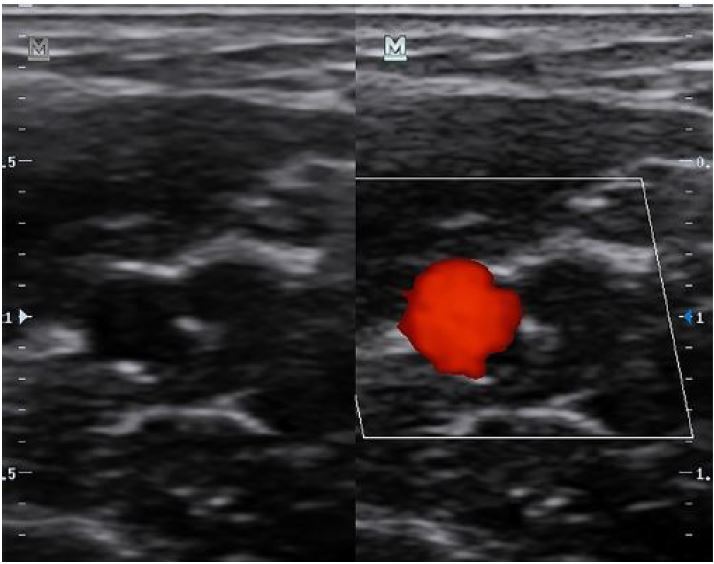
Differentiation of vein from artery using color flow. The right image demonstrates pulsations from the artery.

The first step in placing an ultrasound-guided PIV is to find an appropriate vein to cannulate. With ultrasound guidance, the vein should initially be evaluated by using the probe to apply gentle pressure directly over the vessel (Figure 1, Video 1). Because both arteries and veins will collapse if significant pressure is applied, it is important to apply a small amount of pressure first to assess for pulsatility. If a vein has been confirmed by the above techniques, the provider should then apply full pressure to ensure that no clot is present within the vessel lumen. Providers may also use either color flow or pulsed wave Doppler to verify that the vessel in question is a vein and not an artery (Figure 2, Video 2). Once confirmed, proximal augmentation may be performed to assess for proximal clots that may prevent successful use of the PIV line. To perform proximal augmentation, the provider or patient should squeeze the arm proximal to the proposed PIV insertion site and evaluate for backflow of blood through the vein using color flow Doppler. If the flow is compromised, a different vein should be selected for cannulation. It is important to note that no studies have formally evaluated whether this technique aids in PIV insertion.

The vein should also be measured with respect to both the diameter and depth from the skin surface. Studies have demonstrated that moderate-depth vessels (0.3–1.5 cm from the surface) are significantly easier to cannulate than vessels that are less than 0.3 cm or greater than 1.5 cm from the surface.^32^,^33^ Additionally, Witting et al. demonstrated that vessels greater than 0.4 cm in diameter had a much higher success rate than those less than 0.4 cm in diameter.^33^ While vessel diameter has not been associated with length of PIV sustainability, vessels less than 1.2 cm from the surface have been correlated with significantly longer sustainability of the PIV.^34^ These studies suggest that larger vessels closer to the surface will have the best chance of successful, continued access.

After measuring the diameter and depth of the vein, its course should then be traced with ultrasound to identify the path of the vessel in both short and long axis. Short axis will allow the vessel to be traced in order to identify the direction of the vein and ensure that it remains straight. Long axis will allow for assessment of the presence of valves in close proximity to where the catheter will be placed.^14^ The vessel can be externally marked at the beginning and end of the anticipated catheterization path to assist in following the track of the vessel. However, external marking has not been shown to improve success rates.^35^

When choosing a catheter length, shorter catheters have been demonstrated to have a faster time to cannulation than longer catheters.^36^ However, longer catheters have a lower risk of catheter failure.^36^ Shorter catheters may not have a sufficient length within the actual vessel lumen, thus leading to easier catheter dislodgement and early failure of the PIV.^11^ Longer catheters will allow a greater length of tubing to be within the vessel lumen, which should maintain the catheter within the vessel regardless of patient movement.

When determining which catheter length is necessary, one must consider the total distance that the catheter will travel to enter the vein, rather than just the distance from the vein to the skin surface. This distance is determined using the Pythagorean theorem. Assuming a depth of 1.0 cm with needle insertion at a 45-degree angle, so that the site of the vessel entry is 1.0 cm past the site of skin entry, the provider would actually need to travel 1.4 cm to reach the vein. Based upon existing PIV lengths, the provider should use a catheter that is 2.5 cm or longer to ensure that at least 1 cm of the catheter is securely within the vein.^11^ The table provides a list of recommended catheter lengths based upon the distance from the skin surface to the vein. Of note, if the provider uses a shallower angle, a longer catheter length may be required. For example, if the same vein is 1.0 cm deep and a 30-degree angle is used, the distance to the vein will be 2.2 cm and a longer PIV would be required than in the first example.

There are several options for vein selection when placing an ultrasound-guided PIV. Often, providers use the basilic or deep brachial veins. The basilic vein offers the advantage of being more superficial and separated from the surrounding arteries and nerves. The deep brachial is almost universally present, but is much deeper and in close proximity to the artery and nerve. Consequently, when cannulating the deep brachial vessel, it is important to ensure that sufficient catheter is within the vein and advise patients to minimize arm movements after placement. One study demonstrated that the basilic vein was associated with an improved success rate compared with the deep brachial.^37^ Another study found a significantly higher rate of extravasation in deep brachial veins than in other antecubital veins.^38^ While the focus is often on upper-extremity veins, providers should also consider lower-extremity veins, such as the saphenous vein, which is relatively superficial and separated from surrounding nerves and arteries.^39^

Newer studies have suggested performing ultrasound-guided cannulation of the internal jugular vein using a peripheral intravenous catheter in patients with very limited access.^40^–^44^ While sterile technique (including sterile gloves and a probe cover) is recommended, the catheter is typically treated as a peripheral line after placement. This line has been suggested to be superior to central venous access due to speed of placement and lower risk of complications (e.g., needle injury, catheter malposition, etc.).^40^–^44^ However, the peripheral internal jugular (PIJ) line also carries inherent risks. Given the proximity to central access, providers must be careful to avoid introducing an infection into the central bloodstream. Therefore, a bio-occlusive dressing should be used, and it is not recommended to perform wire exchange through the PIJ to convert it into a central line.^41^ Similar to other PIV, infiltration is a risk with the PIJ and an appropriate catheter length should be chosen to reduce this risk.^41^

## BEST PRACTICE RECOMMENDATIONS

1. Multiple techniques have been used to select a vein for cannulation. The authors recommend vessel compression as the primary technique with color flow Doppler, pulse wave Doppler, or proximal augmentation as supplemental techniques.
2. Veins should be selected that are 0.3–1.5 cm from the skin surface with a diameter greater than 0.4 cm.
3. Catheter length should be selected based upon anticipated distance to the vein to ensure that a sufficient portion of the catheter will remain in the vessel.
4. The deep brachial vein has a higher failure rate and should be avoided when more superficial veins are available.
5. There is limited evidence supporting the ultrasound-guided peripheral internal jugular vein line. Further studies are needed before routine use.

## TECHNIQUE

The most common technique used for the placement of an ultrasound-guided PIV is the short-axis (e.g., transverse or out-of-plane) approach. In this view, the vein will be visualized in cross-section and the needle followed until it enters the vein. With this approach, it is essential that the transducer be advanced in sync with the needle tip, as both the needle tip and shaft may appear similar (Figure 3). While the short-axis approach has been suggested to be faster and easier than the long-axis approach (particularly among more novice sonographers), it may be associated with increased risk of injury to the posterior vessel wall.^45^–^47^

**Figure 4:**
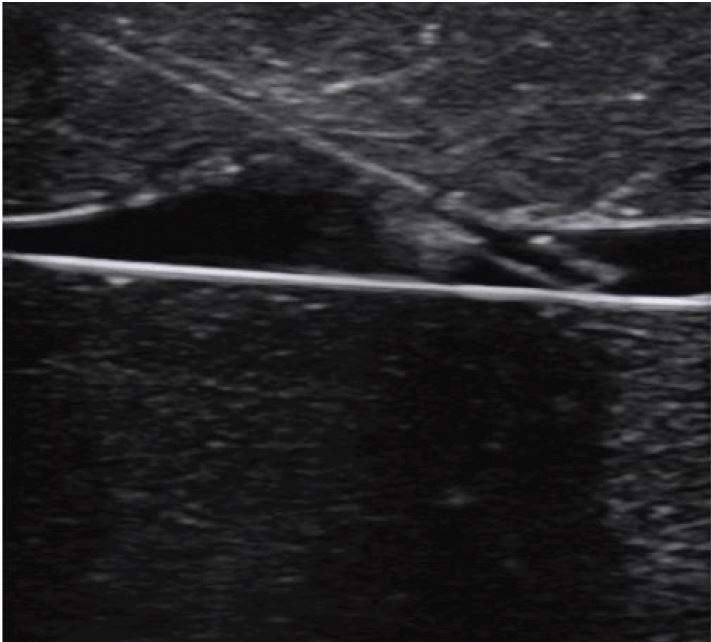
Ultrasound image of peripheral intravenous line in long-axis orientation on a phantom model.

The second most common technique is the long-axis (e.g., in-plane) technique. With this approach, the entire length of the vessel and needle will be visualized (Figure 4). Prior to inserting the needle, one must ensure that the entire length of the vessel is visualized. In the long-axis view, veins may appear similar to arteries. Therefore, prior to needle insertion, one should confirm that the visualized vessel is a vein, using one of the aforementioned techniques. When advancing the needle, it is important that both the needle and vessel remain in the same plane. Because of this, it can be challenging for some sonographers to perform in real patients. The advantage of this technique is that the entire needle is visualized, thereby reducing the risk of posterior wall injury, while ensuring that sufficient length of catheter has entered the vein for successful advancement.

A newer technique derived from the central line literature is the oblique approach.^48^–^50^ This is considered by some to be the best of both approaches.^48^–^50^ This technique involves obtaining the short-axis view and then rotating the transducer 45 degrees into an oblique angle to increase the surface area (and, consequently, the visualization) of the needle. The user benefits from the ability to better visualize the location of the needle with respect to nearby structures, while also having improved needle visualization. Further studies are needed to assess this in PIV placement prior to routine use.

While novice sonographers often prefer the short-axis approach, the long-axis approach allows better needle tip visualization and less risk of posterior wall puncture.^45^–^47^,^51^ Long axis is similarly favored in techniques such as nerve blocks, where accuracy of the needle tip carries similar importance.^52^ To minimize damage to surrounding structures, the authors recommend identifying vessels in the short axis and then converting to long axis for needle insertion.

**Figure 5:**
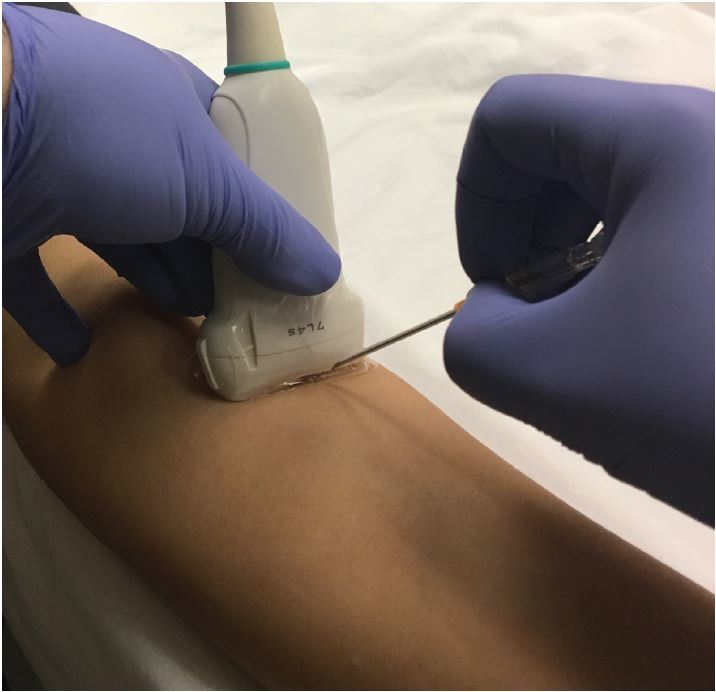
Ideal hand position for ultrasound-guided peripheral intravenous line placement.

Regardless of which technique is used, it is important to avoid accidental compression of the vein during the placement attempt. As patients are often intravascularly depleted and veins are easily compressible, small amounts of pressure may compress or collapse the vein, making cannulation more difficult. This can be avoided by using the palm of the hand or an extended finger to apply pressure and stabilize the hand at a more distant location (Figure 5).

Another common challenge is advancement of the catheter in the short-axis approach. While providers can often obtain initial vessel access with the needle, subsequent threading of the catheter can pose problems. After entering the vessel, the provider should lower the angle of the needle and advance further, while keeping the needle tip in the center of the vessel on ultrasound (Figure 3B). This should be continued, alternating probe and catheter advancement while progressively lowering the angle of the catheter, until the entire length of the catheter is in the vessel and the catheter hub is abutting the skin (Video 3). Using this technique ensures that the maximal length of the catheter is safely inside the vessel, reducing the risk of catheter misplacement or dislodgement.^53^

An additional strategy is to use the Seldinger technique.^54^–^57^ This technique is commonly used for central venous and arterial lines, though not commonly used for peripheral veins. Mahler et al. demonstrated high success rates using this modality in an ED setting.^56^ In cases where a longer catheter with a guidewire is not available, Mills et al. describe a different technique by which initial access is obtained and then the PIV is replaced with a longer, more sustainable PIV, using a guidewire for catheter exchange.^57^

## BEST PRACTICE RECOMMENDATIONS

1. The long-axis approach should be used, when possible, to reduce the risk of posterior vessel wall injury.
2. The authors recommend avoiding compression of the vein by applying pressure distally with the palm or fifth finger.
3. The Seldinger technique may be used to facilitate placement.

## CONFIRMATION

**Figure 6:**
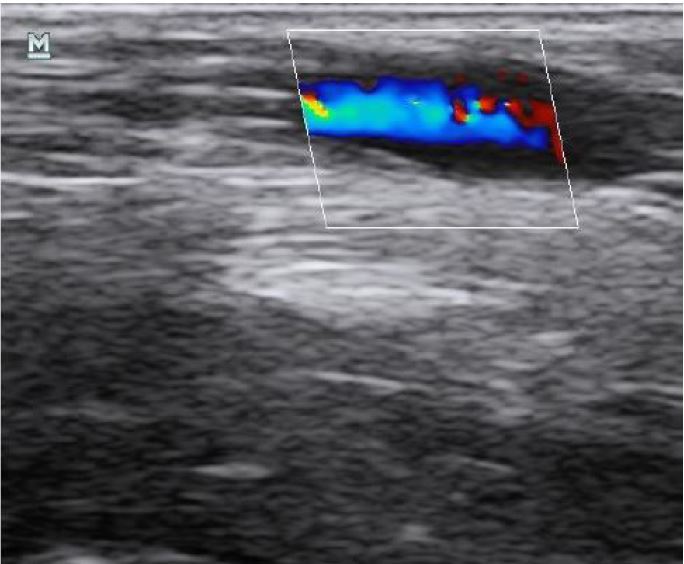
Positive “saline flush test” with color flow Doppler.

Once the PIV has been fully advanced, it is important to confirm placement. While many providers rely upon blood return and the ability to inject saline without palpable soft tissue swelling, ultrasound may be a valuable adjunct for confirming placement. One technique for confirming placement is to visualize the catheter in long axis, ensuring that the entire length of the catheter is within the vessel. This can be further assessed by infusing 5–10 mL of normal saline and visualizing the bubbles appearing within the vessel (e.g. “saline flush test”) (Video 4).^58^–^61^ Color flow can also be added to enhance visualization (Figure 6, Video 5).

## BEST PRACTICE RECOMMENDATIONS

1. Placement may be confirmed by using the ultrasound to visualize the entire length of the catheter within the vessel.
2. Normal saline solution may be infused to further assess proper PIV placement.

## CHALLENGES AND LIMITATIONS

As with most ultrasound procedures, there is operator variability in skill sets. Currently, there is no consensus on the number of observed placements required to determine competency, with studies ranging from 5 – 25 attempts.^14^, ^62^–^66^ Witting et al. found that providers who had placed more than 20 ultrasound-guided PIV had higher success rates than those who had placed less than 20.^33^ More data is needed to determine the number of ultrasound-guided PIVs to become competent in this modality.

Additionally, similar to blind PIVs, if there is insufficient or minimal catheter within the vessel lumen, it may dislodge with arm movements, resulting in loss of venous access and extravasation of infused solution. This risk can be reduced by using longer PIVs and advancing the entire length of the PIV under ultrasound guidance, as discussed above. Finally, one should make sure to properly clean all involved areas and maintain sterility throughout the procedure. While studies have demonstrated no increased infection risks with the use of ultrasound, the addition of the ultrasound machine provides a further potential source for infection if not properly cleaned.

## CONCLUSION

This paper provides a review of the existing data on ultrasound-guided PIV placement combined with suggestions to enhance successful placement and confirmation. After reading this paper, it is the authors’ intention that the reader will have new strategies and troubleshooting techniques for his or her next ultrasound-guided PIV attempt.

## Figures and Tables

**Figure 3A:**
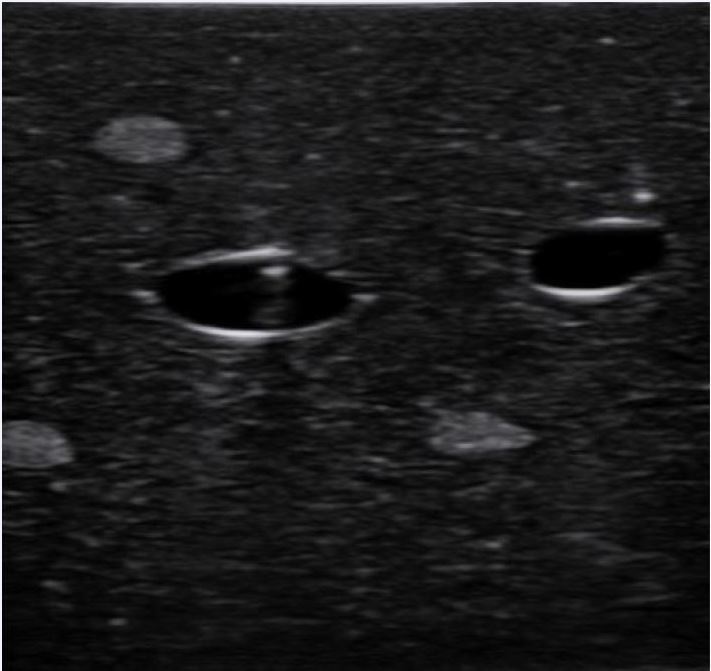
Ultrasound image of needle shaft in short axis on a phantom model.

**Figure 3B:**
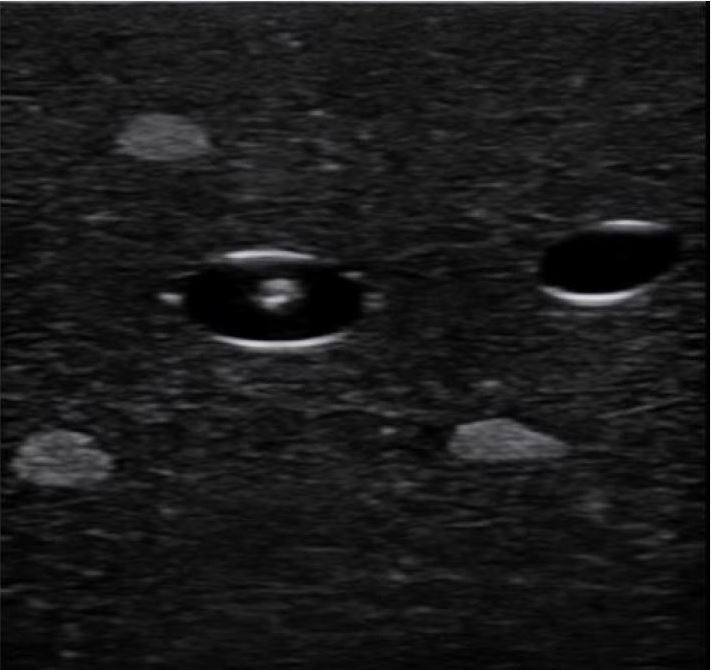
Ultrasound image of needle tip in short axis on a phantom model. Note that the needle tip is slightly more echogenic than the needle shaft.

**Video 1:** Ultrasound video demonstrating vein compression.

**Video 2:** Ultrasound video demonstrating the use of color Doppler to differentiate a vein from an artery.

**Video 3:** Ultrasound video demonstrating needle advancement into a vein in an ultrasound phantom model.

**Video 4:** Ultrasound video demonstrating a positive saline flush test.

**Video 5:** Ultrasound video demonstrating a positive saline flush test with color Doppler.

**Table:** Recommended catheter lengths based upon depth of vein using a 45-degree insertion angle.

Depth of vein in cm	Horizontal distance from the vein for insertion in cm	Total distance to vein in cm	Recommended catheter length in cm (in)
0.5	0.5	0.7	1.9 (0.75)
1.0	1.0	1.4	2.5 (1.0)
1.5	1.5	2.1	3.12 (1.25)
2.0	2.0	2.8	4.4 (1.75)
